# Influence of changes in foot morphology and temperature on bruised toenail injury risk during running

**DOI:** 10.1038/s41598-024-51826-w

**Published:** 2024-01-21

**Authors:** Yang Song, Xuanzhen Cen, Dong Sun, István Bíró, Zhuqing Mao, Yufei Fang, Yaodong Gu

**Affiliations:** 1 Research Academy of Medicine Combining Sports, Ningbo No.2 Hospital, Ningbo, China; 2 Faculty of Sports Science, Ningbo University, Ningbo, China; 3 Department of Biomedical Engineering, Faculty of Engineering, The Hong Kong Polytechnic University, Hong Kong SAR, China; 4 Doctoral School on Safety and Security Sciences, Óbuda University, Budapest, Hungary; 5 Faculty of Engineering, University of Szeged, Szeged, Hungary

**Keywords:** Musculoskeletal system, Risk factors, Biomedical engineering

## Abstract

Despite runners frequently suffering from dermatologic issues during long distance running, there is no compelling evidence quantitatively investigating their underlying injury mechanism. This study aimed to determine the foot morphology and temperature changes during long distance running and reveal the effect of these alterations on the injury risk of bruised toenail by measuring the subjective-perceived hallux comfort and gap length between the hallux and toebox of the shoe. Ten recreational runners participated in the experimental tests before (baseline), immediately after 5 and 10 km of treadmill running (12 km/h), in which the foot morphology was measured by a 3D foot scanner, the foot temperature was detected by an infrared camera, the perceived comfort was recorded by a visual analogue scale, and the gap length in the sagittal plane was captured by a high-speed camera. Ball width became narrower (106.39 ± 6.55 mm) and arch height (12.20 ± 2.34 mm) was reduced greatly after the 10 km run (*p* < 0.05). Foot temperature increased significantly after 5 and 10 km of running, and the temperature of dorsal hallux (35.12 ± 1.46 °C), dorsal metatarsal (35.92 ± 1.59 °C), and medial plantar metatarsal (37.26 ± 1.34 °C) regions continued to increase greatly from 5 to 10 km of running (*p* < 0.05). Regarding hallux comfort, the perceived scores significantly reduced after 5 and 10 km of running (2.10 ± 0.99, *p* < 0.05). In addition, during one running gait cycle, there was a significant increase in gap length at initial contact (39.56 ± 6.45 mm, *p* < 0.05) for a 10 km run, followed by a notable decrease upon reaching midstance (29.28 ± 6.81 mm, *p* < 0.05). It is concluded that the reduced ball width and arch height while increased foot temperature during long-distance running would exacerbate foot-shoe interaction, potentially responsible for bruised toenail injuries.

## Introduction

As one of the convenient and low-cost forms of exercise, running has attracted extensive participation by people of all age groups around the world^1,2^. The possibility of obtaining multiple benefits such as weight loss, improved cardiovascular health, and stress relief makes it no surprise that the number of runners and running events has grown progressively during recent years^1,3,4^. More participants tend to run long distances in order to gain further benefits from a long-term perspective. However, wide participation in running, especially the prolonged distance, has been found to be associated with a higher injury rate of lower limbs and feet^5–12^. Among all these running-related injuries, dermatologic issues are more frequently encountered^9–12^. For example, in a review of post-marathon injuries, Mailler-Savage et al.^9^ reported that at least 20% of injuries sustained by marathon runners are related to the skin. While some of the dermatologic injuries that occur during running may seem minor, they can have a significant impact on physical activity and, in some cases, even pose a life-threatening risk^10,12^. Therefore, it is crucial to identify the underlying injury mechanisms responsible for these injuries to mitigate their potential morbidity and enhance overall running safety.

Subungual hematomas, which appear as a collection of blood below the nail plate, are one of the most common types of dermatologic injuries that can bedevil runners after a race^13,14^. These injuries can lead to significant subjective-perceived discomfort due to the pressure forces that develop at the nail bed and may cause temporary limitations of activities. In some cases they could further result in long-term complications such as secondary fungal infections and nail plate deformities^13,14^. It has been widely documented that the repetitive contact between the nail bed and toebox of the shoe is responsible for developing subungual hematomas and numerous factors such as poor shoe fit, steep terrain, and lower extremity edema play important roles in this process^9,12–14^. However, to the authors' knowledge, most of the prior research were case studies or epidemiological studies based on questionnaires while there is currently limited quantitative research on this topic. Experimental analysis can offer more accurate information in terms of injury mechanisms and protection strategies. For example, some studies have quantitatively explored the pathomechanics of other skin injuries such as foot blisters, and developed a laboratory-based blister creation model for the quantification of changes in risk of blister^15,16^. From the perspective of the foot itself, it must be noted that foot morphology and foot temperature should be considered as key intrinsic factors that may exacerbate subungual hematomas^17–21^. On one hand, the shape of the foot is not static and can vary depending on individual characteristics. For example, foot shape morphs during dynamic running or walking situations, and its morphology variations may further result in shoes being ill-fitted, while ill-fitted footwear has been shown to be related to dermatologic problems^17,18,22^. On the other hand, the foot exhibits a sophisticated structure with a high density of sweat glands and intricate blood vessel orientation. Meanwhile, while running, the foot consistently makes contact with the ground, producing substantial mechanical energy that dissipates as heat. As a result, these factors collectively generate a microclimate inside the shoe that can increase the foot temperature, which may further lead to excessive foot-shoe interaction and consequently more repetitive trauma because of the sweat that was induced by high temperature^23–25^. Nevertheless, no direct evidence has been reported concerning how foot morphology and temperature changes would affect the risk of toenail injuries during prolonged running activities.

**Figure 1:**
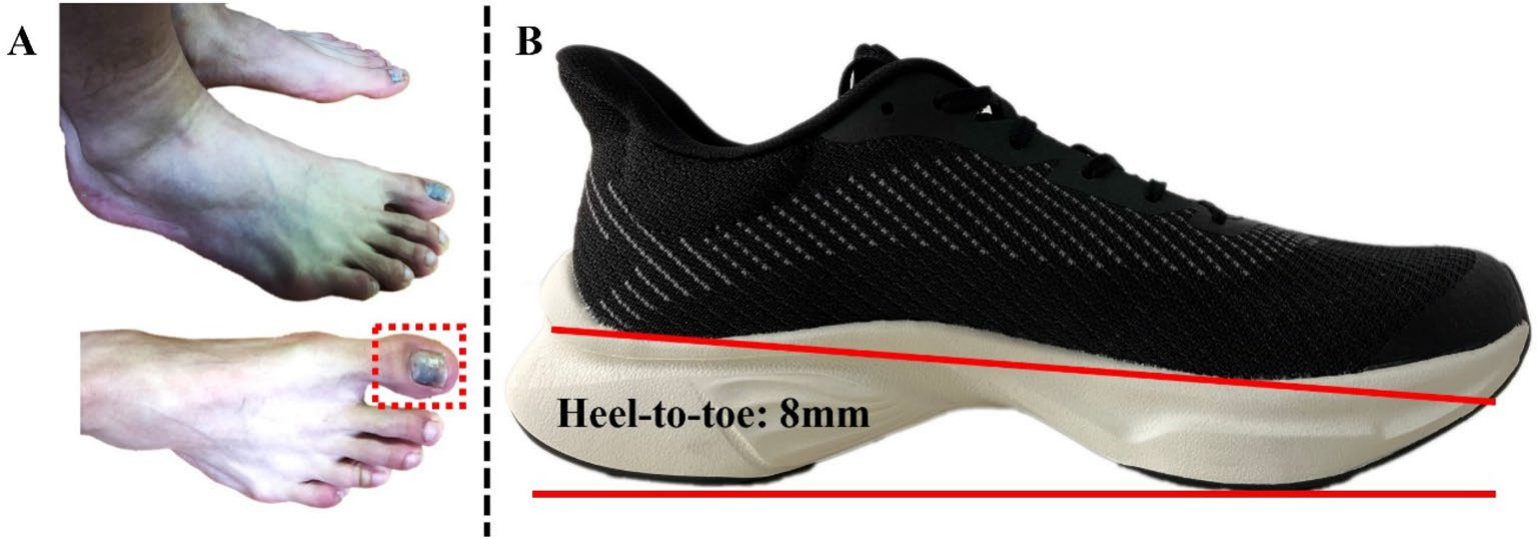
Illustration of the bruised toenail (**A**) and running shoes (**B**).

**Figure 2:**
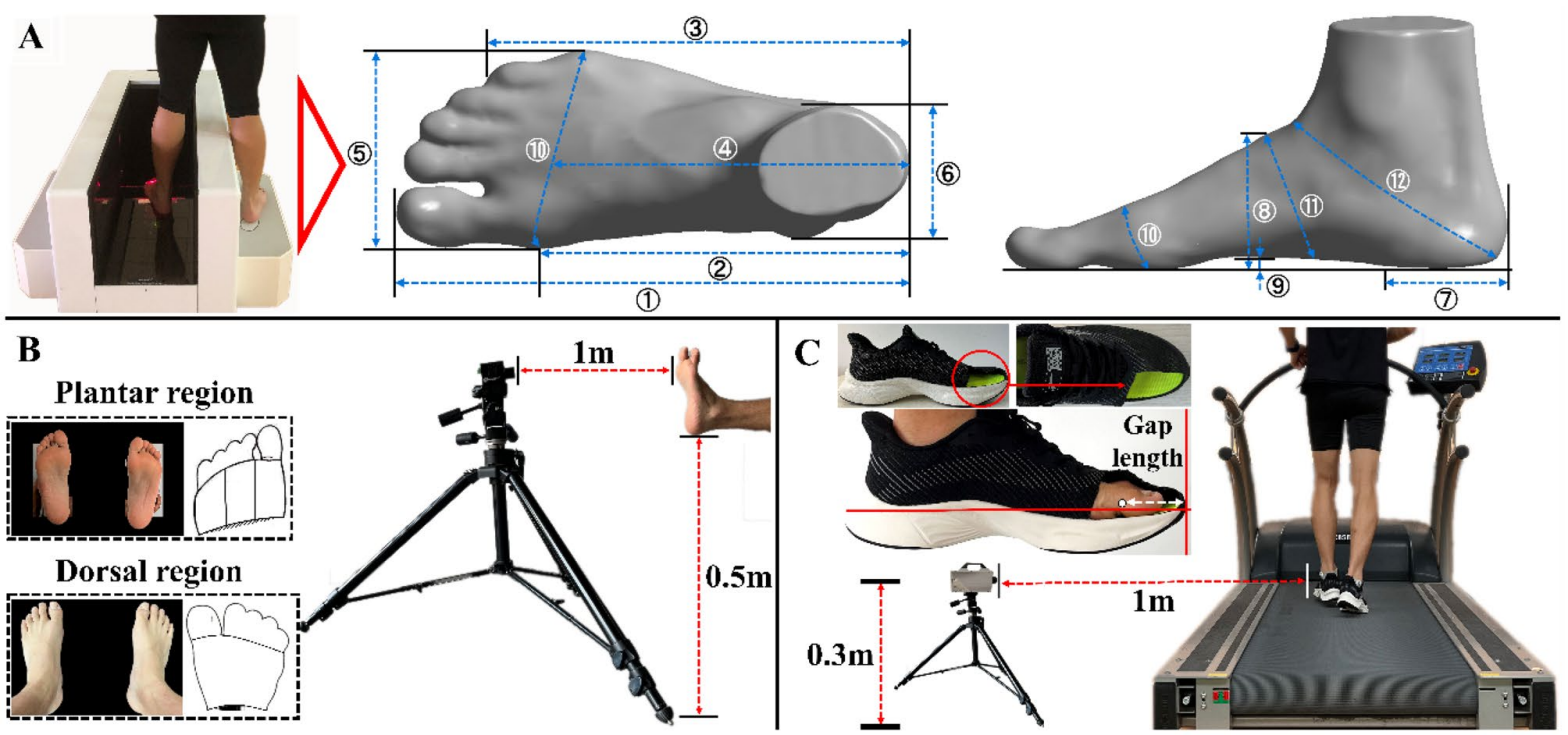
Illustration of data collection setup, foot morphology (**A**), foot temperature (**B**), and gap length between the hallux and toebox of the shoe (**C**). Note: ①, foot length; ②, arch length; ③, heel to fifth toe length; ④, mid-ball to heel length; ⑤, ball width; ⑥, maximal heel width; ⑦, maximal heel location; ⑧, dorsal height; ⑨, arch height; ⑩, ball girth; ⑪, instep girth; ⑫, short heel girth. The subareas for foot temperature measurement include hallux, other toes, medial metatarsal, central metatarsal, lateral metatarsal, dorsal area of hallux, dorsal area of other toes, and dorsal area of metatarsal.

Bruised toenail, also known as Jogger’s toe, is a typical form of subungual hematomas found on the nail plate of the toe^26^. According to findings from Mailler-Savage et al.’s review, the longest toes, particularly the hallux, are most commonly affected. The entire hallux would experience significant trauma during running, leading to approximately 0.1–14% of runners reporting toenail injuries on marathon day^9^. Despite that numerous external variables would affect the incidence of bruised toenail, in this study we aimed to quantitatively determine the changes in the foot itself during a 10 km running, including an interval test after 5 km, investigate the influence of the altered foot morphology and temperature characteristics in the cause of bruised toenail under the case of regular long-distance running by measuring the subjective-perceived comfort of hallux and gap length between the hallux and toebox of the shoe. Based on the findings of previous reports, we hypothesized that following a long run, (1) Foot shape, particularly the ball width and arch height, would notably decrease, alongside a significant overall reduction in foot temperature. (2) These changes in foot morphology and temperature would correspond with reduced perceived comfort and increased foot-shoe interaction (Figs. 1 and 2).

## Results

### Foot morphology and temperature

In terms of foot morphology, only arch height and ball width altered significantly throughout the running test (*p* < 0.001 and *p* = 0.02, Table 1). As shown in Fig. 3, after 10 km of running, arch height was reduced significantly when compared to 5 km (*p* = 0.001) and baseline (*p* = 0.002) conditions, and ball width was also decreased in statistics versus baseline (*p* = 0.039).

**Table 1:** Foot morphology and temperature analysis under baseline and after 5 km and 10 km of running.

	Baseline	5 km	10 km	One-way repeated measures ANOVA		
				F-value	η_*p*_^2^	*p*-value
Morphology (mm)						
①Foot length	249.04(7.13)	250.64(8.12)	250.97(7.05)	0.88	0.09	0.39
②Arch length	176.46(5.33)	178.54(7.00)	177.84(7.75)	0.53	0.06	0.60
③Heel to fifth toe length	200.80(8.39)	200.82(10.53)	201.23(11.35)	0.03	0.01	0.97
④Mid-ball to heel length	209.87(6.28)	210.73(7.16)	210.90(7.18)	0.23	0.03	0.80
⑤Ball width	107.82(6.63)	106.77(6.68)	106.39(6.55)	8.30	0.48	0.02
⑥Maximal heel width	59.13(1.82)	59.52(1.78)	58.51(2.67)	1.48	0.14	0.26
⑦Maximal heel location	61.58(5.61)	61.76(5.56)	60.94(4.78)	0.39	0.04	0.68
⑧Dorsal height	57.09(3.81)	57.65(3.10)	57.18(2.81)	0.44	0.05	0.55
⑨Arch height	13.12(2.58)	12.88(2.47)	12.20(2.34)	26.11	0.74	< 0.001
⑩Ball girth	233.16(6.60)	232.26(6.08)	231.01(6.25)	1.89	0.17	0.20
⑪Instep girth	241.43(8.84)	242.68(9.20)	243.27(8.31)	1.01	0.10	0.38
⑫Short heel girth	320.36(6.29)	321.10(7.82)	319.28(9.83)	0.73	0.08	0.43
Temperature ( °C)						
Hallux	22.66(1.00)	33.47(1.54)	32.53(1.52)	271.36	0.97	< 0.001
Other toes	22.69(1.08)	34.62(1.99)	34.24(2.59)	343.49	0.97	< 0.001
Medial metatarsal	26.46(1.14)	35.24(1.18)	37.26(1.34)	166.40	0.95	< 0.001
Central metatarsal	26.74(0.75)	36.17(1.24)	37.24(1.57)	195.96	0.96	< 0.001
Lateral metatarsal	25.34(1.29)	34.73(1.24)	34.81(2.75)	135.81	0.94	< 0.001
Dorsal area of hallux	22.72(1.04)	33.84(1.85)	35.12(1.46)	415.15	0.98	< 0.001
Dorsal area of other toes	22.48(1.54)	34.34(2.54)	34.27(2.24)	371.92	0.98	< 0.001
Dorsal area of metatarsal	25.27(1.48)	34.24(1.13)	35.92(1.59)	199.67	0.96	< 0.001

**Figure 3:**
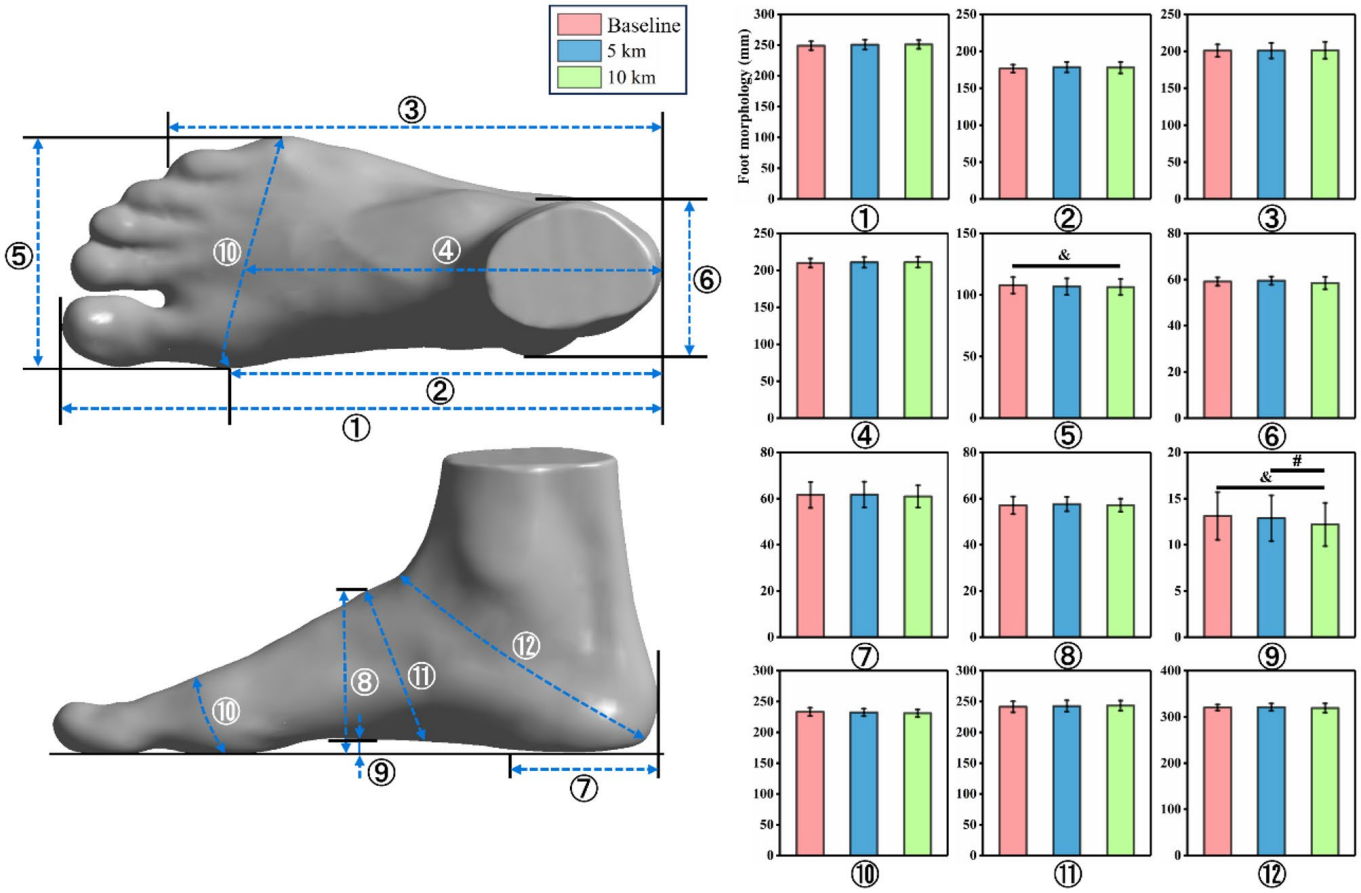
Column chart of the Bonferroni comparisons for foot morphology under baseline and after 5 km and 10 km of running. Note: ①, foot length; ②, arch length; ③, heel to fifth toe length; ④, mid-ball to heel length; ⑤, ball width; ⑥, maximal heel width; ⑦, maximal heel location; ⑧, dorsal height; ⑨, arch height; ⑩, ball girth; ⑪, instep girth; ⑫, short heel girth. * represents a significant difference between baseline and 5 km conditions; & represents a significant difference between baseline and 10 km conditions; and # represents a significant difference between the 5 km and 10 km conditions.

**Figure 4:**
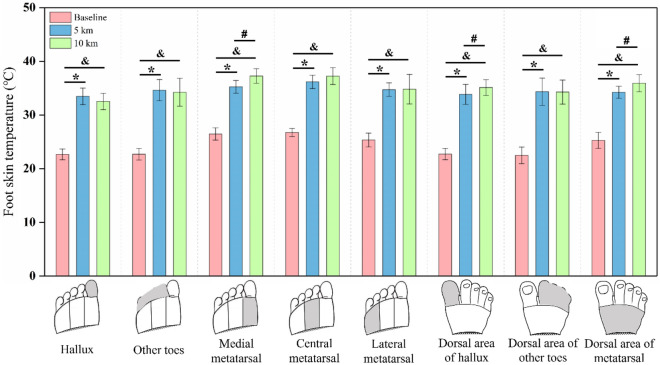
Column chart of the Bonferroni comparisons for foot temperature under baseline and after 5 km and 10 km of running. * represents a significant difference between baseline and 5 km conditions; & represents a significant difference between baseline and 10 km conditions; and # represents a significant difference between the 5 km and 10 km conditions.

For all the forefoot anatomical regions, foot skin temperature exhibited statistical differences among the three conditions with *p* < 0.001 (Table 1). Compared with the baseline, it was found that running (5 km and 10 km) led to significantly higher skin temperature (*p* < 0.001, Fig. 4). Moreover, the temperature of dorsal hallux, dorsal metatarsal, and medial plantar metatarsal regions after 10 km running were also significantly greater than the 5 km (*p* = 0.04, *p* = 0.043, and *p* = 0.014, Fig. 4).

### Subjective-perceived comfort and gap length

ANOVA analysis showed that the VAS scores were statistically different among the three conditions (*p* < 0.001) (Table 2). Compared with the baseline, both 5 km and 10 km significantly increased the hallux VAS levels (*p* = 0.001 and *p* < 0.001). However, no significant difference was presented between 5 and 10 km (*p* > 0.05).

**Table 2:** Perceived VAS scores and gap length analysis under baseline and after 5 km and 10 km of running.

	Baseline	5 km	10 km	One-way repeated measures ANOVA		
				F-value	η_*p*_^2^	*p*-value
*VAS*						
Hallux	0.00(0.00)	1.60(0.84)	2.10(0.99)	29.81	0.77	< 0.001
Gap length (mm) (Running distance)						
Initial contact	38.58(5.90)	38.34(5.27)	39.56(6.45)	4.81	0.09	0.01
Midstance	31.32(7.46)	31.44(6.82)	29.28(6.81)	17.84	0.27	< 0.001
Toe-off	38.23(5.67)	37.97(4.78)	38.17(5.81)	0.22	0.01	0.80
*Gap length (mm) (Stance phase)*	Initial contact	Midstance	Toe-off			
Baseline	38.58(5.90)	31.32(7.46)	38.23(5.67)	175.07	0.78	< 0.001
5 km	38.34(5.27)	31.44(6.82)	37.97(4.78)	253.52	0.84	< 0.001
10 km	39.56(6.45)	29.28(6.81)	38.17(5.81)	396.624	0.89	< 0.001

The calculated gap length between the hallux and toebox of the shoe showed significant differences both in one stance cycle (*p* < 0.001) and throughout the running test (initial contact: *p* = 0.01, midstance: *p* < 0.001) (Table 2). As shown in Fig. 5A, during the stance phase, gap length at the midstance instant reduced significantly when compared to initial contact (*p* < 0.001) and toe-off (*p* < 0.001) in all conditions. It was also found that gap length at the toe-off instant showed a statistical decrease compared to initial contact at 10 km (*p* < 0.001). On the other hand, after 10 km of running (Fig. 5B), gap length at initial contact instant showed a significant increase compared to 5 km (*p* = 0.018), while gap length at midstance instant reduced with significance versus both baseline and 5 km conditions (*p* < 0.001).

**Figure 3:**
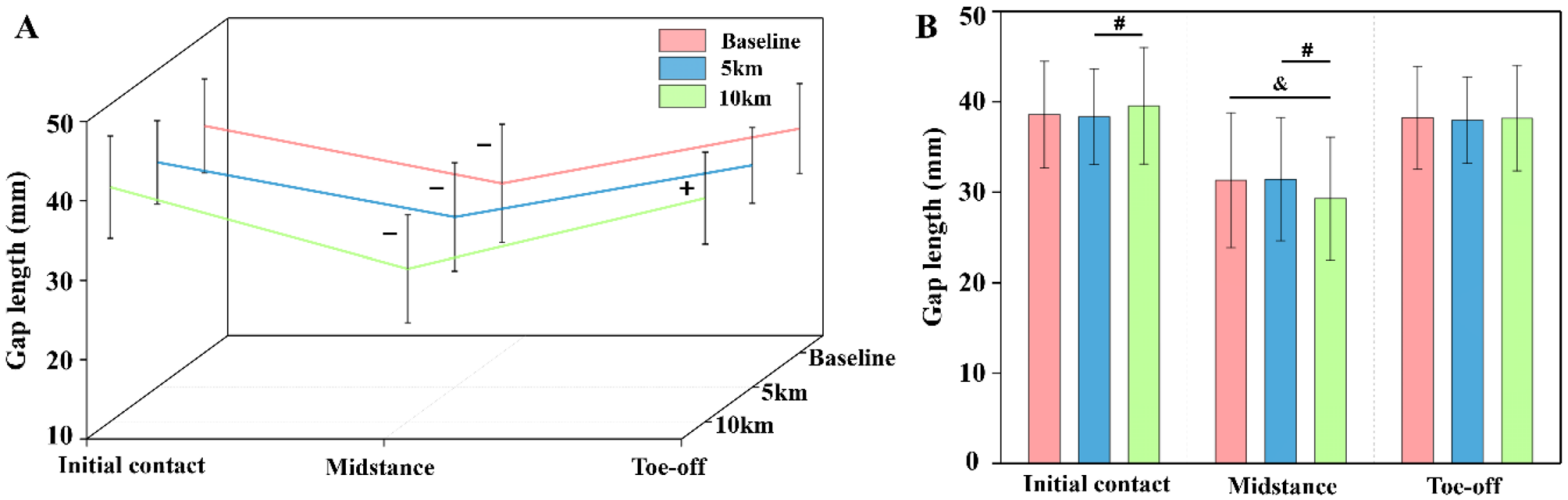
Column chart of the Bonferroni comparisons for gap length under baseline and after 5 km and 10 km of running, gap length comparisons in one stance phase (**A**), and gap length comparisons throughout the running test (**B**). * represents a significant difference between baseline and 5 km conditions; & represents a significant difference between baseline and 10 km conditions; and # represents a significant difference between the 5 km and 10 km conditions.

## Discussion

This study set out to investigate the underlying injury mechanism of bruised toenail during long distance running by measuring foot temperature and morphology alterations, perceived hallux comfort differences, and gap length changes between the hallux and toebox of the shoe after a continuous 5 and 10 km of running when compared to baseline. Consistent with our first hypothesis, it was found that ball width significantly reduced while arch height was reduced greatly after the 10 km run. The foot temperature measured in this study increased significantly after 5 and 10 km of running, and the temperature of dorsal hallux, dorsal metatarsal, and medial plantar metatarsal regions continued to increase greatly from 5 to 10 km of running. The second hypothesis of this study was confirmed in the observation that the perceived hallux comfort greatly reduced after 5 and 10 km of running, and the gap length increased significantly at the initial contact while decreased significantly at the midstance after the 10 km run.

The popularity of long distance running has been reported to be accompanied by a high injury risk^6,8,27^. In order to investigate the incidence and associated potential risk factors of running-related injuries, Van Gent et al.^27^ further divided the determinants into several categories, which include systemic factors (e.g., age, weight, and height), lifestyle factors (e.g., smoking and drinking alcohol), health factors (e.g., injury history), and running related factors (e.g., training frequency, distance, and shoe use), etc. However, little research has considered the effects of foot shape/temperature variations on running-related injuries (e.g., dermatologic injuries) during long distance running.

In this study, the ball width of the foot was found significantly reduced after 10 km of running, which is in accordance with the findings reported by Mei et al.^17^. In their study, Mei et al.^17^ also observed ball girth and foot volume reduction after 20 km of running and they speculated that these foot shape changes could further contribute to more space, especially for the forefoot region, and encourage greater friction between foot and shoe interface since they became less conforming to each other. Despite only 10 km of running test being conducted, the reduced ball width and perceived hallux comfort while increased foot-shoe interaction observed in our study directly confirm the above-mentioned speculation, which may potentially increase the risk of bruised toenail injury. In addition, our study also observed a decreased arch height after 10 km, partly consistent with previous studies since they only detected the significance at 20km^17,28^. This variation may be explained by the subject heterogeneity and/or different running interfaces among studies. Nevertheless, the arch height reduction could further increase the foot ambulation in the sagittal plane and let the toenails take more brunt of the impact with the toebox of the shoe, which may also explain the significantly higher temperature observed at the dorsal hallux and dorsal metatarsal regions in our study.

It was previously indicated that there is a moderate positive correlation between changes in foot temperature and contact load^27,28^. In our study, we observed significantly higher skin temperatures in all forefoot regions after running when compared to baseline, which in turn indicates the greater contact pressure suffered by the foot during the running test. Meanwhile, the increased skin temperature may also be accompanied by sweat, creating an environment that would exacerbate the foot-shoe interaction and consequently increase the injury risk of toenails^17^. Based on the speculation of previous research^17^, it is also worth noting that the reduced ball width observed in our study may be explained by the loss of sweat in the foot during running. In addition, this study exhibited a continued increase in temperature in the medial metatarsal region after 10 km of running, which was in agreement with previous research reporting running-related plantar pressure alterations^17,31–34^. In these studies, it was found that the peak pressure at the medial metatarsal increased while the peak pressure at the hallux reduced during long distance running, indicating that the stress loading would eventually be transferred to the metatarsal region, which potentially increases the injury risk of the metatarsal. Similarly, based on the above information, it can be deduced that the increased temperature at the dorsal hallux region may be a precursor to the onset of the bruised toenail injury.

The key implication of this study was that we for the first time quantitatively confirmed the potential effects of the altered foot morphology and temperature characteristics during long-distance running on the cause of bruised toenail. Based on these findings, recreational runners are advised to consider adjusting their footwear, particularly around the 10 km mark. This may involve changing to athletic socks that keep the foot dry and cool, ensuring the lacing is tight enough to prevent forward sliding without restricting circulation, and selecting a shoe with a high and long anterior toe box to allow unrestricted toe movement and minor forward slippage. However, it must be clarified that only treadmill running was considered in this study. Long-distance running under other situations such as trail running and road running should be further verified. This study also measured the gap length between the hallux and toebox within one stance phase, which for the first time revealed the pattern of foot-shoe interaction and also the time point that bruised toenail may occur during running from a quantitative research perspective. Specifically, after the initial contact with the ground, the shoe has come to a brief moment stop while the foot has not, as shown in the findings of the significantly reduced gap length at the midstance instant compared to initial contact throughout the running test. It is supposed that this is the moment when the toenails would take impact stress with the toebox of the shoe. To make matters worse, it was found in our study that the 10 km of running contributes to more gap length reduction at the midstance instant, which consequently increases the potential injury risk. Afterward, the foot slid backward by a small amount in order to prepare for the push-off. However, additional stress may be applied to the toenails since the toes need to grip the ground for propulsion during this phase. However, previous studies have proposed that the toes’ dynamic functions would gradually lose during long distance running, as indicated by the shift of the loading from the hallux to the medial metatarsal regions^17,31,33,34^. Thus, it is hard to determine which step is more important or if it is a combination.

While this study represents the initial exploration of the biomechanical mechanisms of bruised toenail injury during long-distance running, it is important to acknowledge its inherent limitations. Firstly, only 10 recreational runners with a history of bruised toenail were included, which may have potentially influenced the findings of this study. Subsequent research with a larger sample size is warranted to further validate our findings. Moreover, there are numerous intrinsic and extrinsic factors that could influence the risk of bruised toenail injury, including gender, running speed, running surface, running strike pattern, shoe selection, and more. However, our study only investigated the potential causes of bruised toenails in male runners during a 10 km treadmill run at a constant speed. The advantage is that the running distance can be precisely controlled and the timing of the test (immediately after a 5 and 10 km run) can be guaranteed. Nevertheless, this limitation must be taken into consideration when interpreting the results of this study. Nonetheless, this study could serve as a preliminary reference for bruised toenail injury prevention and running shoe design.

## Conclusion

In summary, this study for the first time quantitatively investigated the effects of foot morphology and temperature alterations during long distance running on the potential cause of bruised toenail injury. It was found that the reduced ball width and arch height while increased skin temperature of the foot after long distance running were accompanied by hallux discomfort and excessive foot-shoe interaction, suggesting an increased risk of bruised toenail injury.

## Materials and methods

### Participants

The recreational runner was defined based on regular running practice (frequency: ≥ 3times/week, distance: ≥ 20 km/week) and the World Masters Association age grading performance tables (age-graded score < 60%), for more details about age-graded score see Liu et al.’s study^35^. Accordingly, thirty male recreational runners were recruited from the university and local running clubs. However, since our study focused on investigating the suspected mechanism in the cause of bruised toenail, only runners who self-reported previous bruised toenail (hallux) experience during long-distance running or marathon competitions were included (Fig. 1A). Thus, a total of ten male recreational runners (age: 26 ± 3 years, height: 1.72 ± 0.04 m, weight: 64.73 ± 5.68 kg, BMI: 21.99 ± 2.75 kg/m^2^, running experiences: 4.25 ± 1.81 years, running frequency: 3 ± 1 times/week, running distance: 28.70 ± 8.21 km/week) who all experienced bruised toenails on the dominant foot over the past six months participated in this study. All participants were confirmed as right leg-dominant based on the ball-kick test, habitual rearfoot strikers, and had no other lower limb and foot musculoskeletal injuries at least six months before the experiment. In terms of the running shoe, the same one with EVA midsole, rubber outsole, and 8 mm heel-to-toe drop was used in this study (Fig. 1B). All participants preferred the same shoe size of 41 (Europe) and were required to wear the experimental shoe while performing daily exercise for one week (similar intensity and volume) before the test. In addition, the same researcher laced up the running shoes during the test to avoid any influences from external factors.

Written informed consent was obtained from each participant after introducing the test requirements and procedures. This study was performed in compliance with the declaration of Helsinki and was approved by the Ethics Committee of Research Academy of Grand Health at Ningbo University (RAGH20211013).

### Experiment protocol

Prior to the test, participants were required to wear the experiment shoes and run on the treadmill at a speed of 8 km/h for 10 min as a warm-up. The foot morphology, foot skin temperature, subjective-perceived hallux comfort, and gap length between the hallux and toebox were then collected for baseline data. After that, each ran on the treadmill with a speed of 12 km/h and a 0% slope for 10 km in total. The variables mentioned above were collected immediately at the time when they finished the 5 km and 10 km running.

### Data collection and processing

The foot shape data were collected using a 3D foot scanner (Easy-Foot-Scan, OrthoBaltic, Kaunas, Lithuania) with an accuracy of 0.3 mm. Participants had their dominant foot scanned while standing with legs separated at shoulder width. Twelve foot dimensions were measured according to a previously established protocol (Fig. 2A)^36^. The foot skin temperature was recorded by an infrared camera (Magnity Electronics Co. Ltd., Shanghai, P.R. China) with a resolution of 384 × 288 pixels, measurement uncertainty of ± 2 °C or 2%, and noise equivalent temperature difference (NETD) < 0.06 °C. For the plantar region, participants sat down with their legs in a horizontal position and perpendicular to the infrared camera at a distance of 1 m. An anti-reflection panel was placed behind the feet to minimize any other effects from the surroundings and body^25^. For the dorsal region, participants stood on the panel, and the infrared camera was placed 1 m above the ground (Fig. 2B). The indoor temperature was controlled at 20 °C using an air conditioner, and participants were required to remain barefoot for 10 min to adapt to the room temperature before the thermographic measurement for baseline^37^. Based on the foot anatomical model, 8 regions of interest (Fig. 2B) were defined to obtain the mean temperature at the skin emissivity factor of 0.98 using ThermoScope v1.2 (Magnity Electronics Co. Ltd., Shanghai, P.R. China), which includes (1) hallux, (2) other toes, (3) Medial metatarsal, (4) central metatarsal, (5) Lateral metatarsal, (6) dorsal area of hallux, (7) dorsal area of other toes, and (8) dorsal area of metatarsal. Only the forefoot region was considered in our study and the proportion criteria for the delimitation of the regions of interest can be found in our previous study^21^.

The subjective-perceived comfort of hallux was measured using the visual analogue scale (VAS). In this study we considered VAS to be reported in centimeters (i.e., 0–10), with the left end (0) as “no pain” and the right end (10) as “unbearable pain”^38^. Lastly, the gap length between the hallux and toebox of the shoe, defined as the horizontal distance between the phalangeal joint of the hallux and toe cap in the sagittal plane, was collected with a high-speed digital camera (Fastcam SA3, Photron, Japan) at a frequency of 1000 Hz. The 2D video analysis using a high-speed camera has been previously shown to be reliable and valid for assessing running kinematics^39^. As shown in Fig. 2C, the camera was automatically calibrated and positioned on a portable tripod placed 30 cm above the floor and 1 m away from the treadmill. A triangle-shaped hole was cut in the shoe upper and a reflective marker (1.5 mm) was attached to the phalangeal joint of the hallux to allow the camera to capture the hallux motion. Participants were instructed to switch to the modified shoe and run at 12 km/h until 5 complete gait cycles were recorded for each condition. Still-frame images corresponding to three stance instants (initial contact, midstance, and toe-off) were initially extracted from the recorded 2D videos. Subsequently, the gap length was visually quantified through video analysis software (Photron FASTCAM Viewer Ver.3620, Photron, Japan) by measuring the horizontal distance between two points (the reflective point and toe cap). Initial contact was defined as the moment when the shoe makes first contact with the treadmill belt, midstance was defined as the moment when the knee of the swing lower limb was adjacent to the knee of the stance one, and toe-off was defined as the moment when the shoe makes the last contact with the treadmill belt^39^. The same researcher (Y.S.) conducted all procedures and data analyses to ensure consistency.

### Statistical analysis

All statistical analyses were performed with the SPSS 22 (IBM SPSS Inc., Chicago, IL, USA). Prior to analysis, the Shapiro–Wilk test was applied to check the normality of data distribution and Levene’s test for homogeneity of variances was used for homogeneity assessment. One-way repeated-measures analysis of variance (ANOVA) with Bonferroni post-hoc comparison was conducted to determine the differences in the above-mentioned four aspects among baseline, 5 km, and 10 km conditions. The gap length differences among three stance instants (initial contact, midstance, and toe-off) in one gait cycle were also compared using the above statistical method. The effect size was computed to quantify the magnitude statistically using the partial eta squared value (η_p_^2^: small (0.01 < ES ≤ 0.06), medium (0.06 < ES ≤ 0.14), and large (ES > 0.14)^40^. The significance level was set at **p** < 0.05.

## Data Availability

The datasets generated during and/or analyzed during the current study are available from the corresponding author on reasonable request.
